# Surgical site infection following cesarean section in a general hospital in Kuwait: trends and risk factors

**DOI:** 10.1017/S0950268819001675

**Published:** 2019-10-10

**Authors:** W. Alfouzan, M. Al Fadhli, N. Abdo, W. Alali, R. Dhar

**Affiliations:** 1 Microbiology Unit, Department of Laboratories, Safat, Kuwait; 2 Department of Microbiology, Faculty of Medicine, Kuwait University, Safat, Kuwait; 3 Infection Control Directorate, Sabah Area, Kuwait, Kuwait; 4 Department of Infection Control, Farwania Hospital, Kuwait, Kuwait; 5 Department of Community, Environmental and Occupational Medicine, Faculty of Medicine, Zagazig University, Zagazig, Egypt; 6 Department of Epidemiology and Biostatistics, Faculty of Public Health, Kuwait University, Kuwait, Kuwait

**Keywords:** Cesarean section, risk factors, surgical site infection, surveillance

## Abstract

Surgical site infections (SSI) are a significant cause of post-surgical morbidity and mortality. The objectives of this study were to determine the prevalence of SSI and identify risk factors for infections following cesarean section (CS). A prospective study of SSI after CS was carried out from January 2014 to December 2016 using the methodology of the American National Nosocomial Infection Surveillance System. Suspected SSIs were confirmed clinically by the surgeon, and or, by culture. Seven thousand two hundred thirty five CS were performed with an overall SSI prevalence of 2.1%, increasing from 1.7% in 2014 to 2.95% in 2016 (*P* = 0.010). Of 152 cases of SSI, the prevalence of infection was 46.7% in women ⩽30 years and 53.3% in women >30 years (*P* = 0.119). Of 148 culture samples from as many women, 112 (75.7%) yielded growth of microorganisms with 42 (37.5%) of isolates being multi-drug resistant (MDR). Women who did not receive prophylactic antibiotics (35.5%) developed SSI more often than those who did (*P* < 0.0001). These findings suggest that emergency CS and inappropriate antibiotic prophylaxis are risk factors for developing SSI. In the light of the emergence of MDR bacteria there is a need to implement revised prophylactic antibiotic policy as part of antimicrobial stewardship to decrease SSI rates.

## Introduction

The cesarean section (CS) is one of the most common obstetrical surgical procedures. It is performed when clinically indicated to facilitate delivery in complicated cases; hence preventing maternal and perinatal morbidity and mortality [1]. Since 1985, the international healthcare community has considered an appropriate proportion for CS to be between 10–15% of all deliveries [2]. However, a recent WHO report has highlighted that the CS has become increasingly common in both developed and developing countries for a variety of reasons [3–5]. Suggested optimal rates ranging from 5–20% including both minimal desirable levels of emergency CS and those constituting overuse of the procedure (elective CS) [5]. The procedure is associated with short- and long-term risks such as maternal deaths, intensive care admission, blood transfusion and hysterectomy, all of which affect the woman and her child's health as well as future pregnancies. The risks are especially higher in women with limited access to comprehensive obstetric care [6–9]. Both low and high levels of the CS use may result in adverse consequences with an increase in maternal and newborn morbidity and mortality in the former and infection, hemorrhage and surgical complications in the latter, exceeding the risks of vaginal deliveries [7, 9, 10]. Among surgical complications, surgical site infection (SSI) rates range from 3–15% worldwide [11–13], and is defined as infection which occurs within 30 days of a post-surgical procedure involving skin, subcutaneous tissue, soft tissue or any other part of the anatomy [14]. The variation in SSI incidence may reflect differences in population characteristics, risk factors, peri-operative practices and post-discharge surveillance for infection. The risk of developing SSI has significantly decreased in the past three decades as reported from several countries worldwide [6, 15], mainly owing to improvements in hygienic conditions, better use of prophylactic antibiotics and adherence to standard infection control protocols. However, with the global increase in the number of CS performed, especially in middle-income countries, SSI incidence is expected to increase. Although post-CS wound infections are not usually serious, they can cause maternal pain and discomfort, post-surgical morbidity, psychological stress and extended hospital stay, which are associated with high-medical expenditure [16].

To the best of our knowledge, limited information exists regarding the magnitude of SSI after CS in Kuwait. The objective of this study was to assess the prevalence and factors associated with post-CS SSI at Farwania Hospital, one of the major secondary care hospitals in Kuwait.

## Methods

### Study setting

The study was conducted at Farwania Hospital, which is a 1200-bed facility with multiple medical and surgical specialties including Obstetrics and Gynecology.

### Study design

This was a descriptive retrospective study of the CS-SSI surveillance data collected for women who gave birth by CS during 2014–2016. All women who underwent CS were actively monitored for signs and symptoms of SSI during hospital admission and after discharge (during follow up in the outpatient clinic) for 30 days (surveillance period) and the relevant information was recorded in the Kuwait National Health Surveillance System (KNHSS) structured forms by the hospital infection control nurses. Data for this study were retrieved from the completed forms. Phone calls were conducted by infection control nurses to reach patients who could not come to the outpatient clinic for follow up.

SSI was based on the definition proposed by the Centers for Disease Control and Prevention (CDC), Atlanta, GA [17]. SSI was considered as superficial when it involved only skin and subcutaneous tissue of the incision site with a purulent discharge or a surgeon's decision on the diagnosis. A deep incisional infection was defined as a discharging wound with deep tissue involvement and abscess formation, whereas an organ/space infection was a combination of deep incision characteristics with extension beyond the fascial/muscle layers. All patients who underwent CS during the study period at Farwania Hospital were recruited.

The collected patient data included the following variables: age, nationality (Kuwaiti or non-Kuwaiti), whether CS was performed as an emergency or elective surgery, duration of surgery, surgical wound classification (clean, clean contaminated, contaminated and dirty/infected), detection during hospital stay or post-discharge (with or without readmission), days to SSI development, culture results, the American Society of Anesthesiologists (ASA) physical status classification score, duration of surgery and compliance with the institutional protocol of prophylactic antibiotics for CS.

### Statistical analysis

Descriptive statistics of the SSI overall prevalence and by the various patient data variables were provided. Furthermore, the SSI percentage was cross-tabulated by operation type, duration of the surgery, wound class, ASA and risk score. Moreover, the percentage of multi-drug resistant (MDR) organisms (resistant to ⩾3 broad-spectrum drug classes including cephalosporins, quinolones, aminoglycosides among others) was cross-tabulated across the following factors: prophylactic antibiotic administration, wound class, wound category, infection interval (time from date of operation until date of infection), duration of the operation (in minutes) and type of operation. The percentage (i.e., prevalence) of each outcome was compared across the levels of each risk factor, using a 2 × 2 *χ*^2^ or 2 × *n* likelihood ratio *χ*^2^ test, as appropriate, in STATA software version 15 (Stata Corp., College Station, Texas).

## Results

A total of 7235 pregnant women underwent CS during the study period. Of these, SSI was documented in 152 women. The overall prevalence rate was 2.1%, which increased from 1.7% in 2014 to 2.95% in 2016. Comparative data on surgery class, risk and ASA scores, and duration of surgery in infected and non-infected cases are presented in Table 1. No significant difference in these characteristics was found among patients in the two groups (*P* > 0.05). Characteristics of the non-infected group of patients (2280) are presented in Table 2. Clean surgery was observed in 99.5% of these patients as compared with those who underwent emergency surgery (*P* < 0.001). On the other hand, clean contaminated surgery was performed in cases of emergency CS. Almost all the patients (99.3%) developed post-discharge SSI with 149/152 (98%) presenting with superficial incisional SSI. One patient presented with a deep incisional SSI, and two (1.3%) developed an organ/space infection which progressed to the blood stream. The time to SSI development varied from 2–30 days with 113/152 (74.3%) of women presenting with the infection in ⩽15 days post-surgery (Table 3). At the time of presentation of SSI, a swab specimen was collected from the wound for culture and antimicrobial susceptibility testing in 148 of 152 patients. Cultures from 112 patients yielded growth of one, two or three species of organisms in 83, 26 and 3 samples, respectively; no growth was observed for 36 samples. Among 54 patients who did not receive any prophylactic antibiotic, 14 (25.8%) were found to be infected with a MDR organism as compared to 28/98 (28.5%) who had received cefuroxime, gentamicin or metronidazole either as a single drug or in different combinations. Of 42 MDR isolates, 34 (80.9%) were identified as methicillin-resistant *Staphylococcus aureus* (MRSA), and three each as *Klebsiella pneumoniae* (extended-spectrum *β*-lactamase (ESBL)-positive) and *Escherichia coli* (ESBL)-positive (Table 3).

**Table 1.** Comparison of infected versus control women post-CS

	CS		
Characteristic	Infected cases (152)	Controls (2280)^a^	*P* Value
	*n* (%)	*n* (%)	
Surgery class			
Clean	19 (12.5)	196 (8.6)	0.101
Clean contaminated	133 (87.5)	2084 (91.4)	
Risk score			
0	136 (89.5)	2016 (88.4)	0.691
1	16 (10.5)	264 (11.6)	
ASA^b^ score			
1	103 (67.8)	1370 (60.0)	0.151
2	49 (32.2)	904 (39.6)	
3	0	6 (0.3)	
Duration of surgery (minutes ± s.d.^c^)	40.65 ± 14.75	41.1 ± 17.8	0.755

CS: cesarean section.

a Data available for control cases in 2016 only.

b American Society of Anesthesiologists.

c Standard deviation.

**Table 2.** Characteristics of 2280 control group women

	CS		
Characteristic	Elective	Emergency	*P* Value
	*n* (%)	*n* (%)	
Surgery class			
Clean	195 (99.5)	768 (36.9)	0.0001
Clean contaminated	1 (0.5)	1316 (63.1)	
Risk score			
0	815 (40.5)	147 (55.7)	0.0001
1	1199 (59.5)	117 (44.3)	
ASA^a^ score			
1	588 (61.1)	783 (59.4)	0.678
2	373 (38.7)	530 (40.3)	
3	2 (0.2)	4 (0.3)	

a American Society of Anesthesiologists.

**Table 3.** Microbiological characteristics of infected patients (152)

Characteristics	Patients *n* (%)	*P* Value
Antibiotic prophylaxis		
Yes	98 (64.4)	<0.001
No	54 (35.6)	
Days to infection		
⩽15 days	113 (74.3)	<0.001
⩾15 days	39 (25.7)	
Wound culture		
Done	148 (97.4)	<0.001
Positive growth	112 (75.7)	
No growth	36 (24.3)	
Organisms isolated (*n* = 144)		<0.001
No MDRO (102)		
MDRO (42)		
MRSA	34 (30.3)	
*K. pneumonia* (ESBL+)	3 (6.2)	
*E. coli* (ESBL+)	3 (6.2)	
*P. mirabilis* (ESBL+)	1 (0.9)	
*A. baumannii*	1 (0.9)	

MDRO, multi-drug resistant organism; MRSA, methicillin-resistant *Staphylococcus aureus*; ESBL+, extended-spectrum beta lactamase positive.

The SSI prevalence was not significantly (*P* > 0.05) associated with the operation type, duration of the surgery, wound class, ASA, or risk score. The percentage of MDR organisms was significantly (*P* = 0.016) associated with the infection interval (time from date of operation until date of infection). However, the MDR infection percentage was not significantly (*P* > 0.05) associated with prophylactic antibiotic administration, wound class, wound category, duration of the operation, or type of operation.

## Discussion

Since the hospital stay after CS for most women is generally less than 72 h it is imperative to perform post-discharge surveillance for the optimal detection of SSI in these cases as it has been shown that the lack of surveillance in these patients results in under-notification of SSI. In the present study, 99% of the patients with SSI were diagnosed after discharge from the hospital which is consistent with international data showing variable infection rates, defined by CDC diagnostic criteria, depending on the type of post-discharge surveillance implemented [18–22]. Likewise, similar to an earlier study where patients with post-CS SSI were identified as outpatients, SSI cases in the present study who presented in the emergency room or the out-patient department were contacted by the infection control nurse after being notified by the attending physician [23].

The incidence of post-CS SSIs appears to vary with a geographical region with higher rates in under developed countries, as illustrated by an average of 7.3% (range, 1.7–10.4%) among in-patients in Sub-Saharan Africa [24], compared with surveys in European countries conducted from 2008–2013 which reported rates from 1.75–4.78%, which included in-patients and post-discharge patients [25]. However, the median length of post-CS surveillance in the latter studies varied from 2–14 days, making it difficult to have a clear and meaningful inter-comparison. Thus, in order to allow comparison, a fixed duration of follow-up should be appropriate. In previous studies the increasing trend of SSI cumulative incidence was ascribed to superficial SSI because of a better post-discharge detection capacity.

The rate of superficial SSIs found here (98%) is almost the same as reported from Italy (96%) [25] and Brazil (97.7%) [26]. Indeed, complicated SSI was very rare (1.9% of cases) in the present study perhaps mirroring the decline in the number of cases of complex SSI from 0.84% in 2008 to 0.19% in 2011 and 0.15% in 2013 as observed by Ferraro *et al*. [25]. Recently, it has been proposed to exclude superficial SSI and infections diagnosed out of the healthcare setting and consider only complex SSI for surveillance, as the subjective, inconsistent and prone to error diagnosis of superficial infections, especially if self-assessed by the patient, would lead to a wide variability of data reporting and to their unreliability as a proxy of health system quality [27, 28]. Although labour intensive, a 30-day post-discharge surveillance is recommended in order to detect almost all SSI (including complicated infections) in the post-discharge period [29]. Since complex SSI cases would require medical care in the healthcare setting it would be expedient for a standardised surveillance method to be applied under those conditions [29]. In contrast to this view, a study from Brazil reported that for 93% of the women who developed SSI, the infection became apparent in the first 15 days following CS [26]. Since data from similar studies [23, 30, 31] corroborated this finding, the authors suggested that a 30-day follow-up period might prove unnecessary for the detection of post-CS SSI. Besides the number of days used for follow-up post-CS for the detection of SSI, the variability in infection rates also depends on the methodology used for surveillance. Although there is no consensus on the best measure for implementing post-discharge surveillance, telephone contact appears to represent a low-cost technique that requires minimal resources and is widely used. Nevertheless, it has been shown to have a relatively low-positive predictive value (28.7%) for diagnoses made according to patient telephone reports, although the negative predictive value was high (98.2%) when compared with a diagnosis made by an infection control nurse through direct examination of the surgical site in the out-patient setting following discharge from hospital [32].

Multiple factors have been shown to contribute to post-CS SSI. Although several non-CS studies have demonstrated an association between age >65 years and SSI; all women in our study were younger than 45 years of age. However, younger age has been associated with SSI among women who underwent CS although no significant difference in age was demonstrable in our patient population [16, 24].

A high proportion of SSI (25.2%) has been reported in emergency CS when compared to 7.6% in elective cases [33]. In the latter study, also from this region (Oman), 1.5% of SSIs were reported after emergency CS compared to 1.16% in elective cases [33], whereas in our series the proportions were 1.56% after emergency CS *vs.* 0.53% post-elective CS.

Post-CS-SSIs can be prevented or the rates reduced by including appropriate preoperative antibiotic prophylaxis as part of the SSI prevention bundle [34–36]. The antibiotic should be administered 60 min before incision to ensure adequate blood and tissue concentrations throughout the operation [35, 37]. Although a single 1 g intravenous dose of cefazolin [38] is suggested in our local antibiotic therapy guidelines with clindamycin as an alternate drug and metronidazole if there is history of premature rupture of membranes (PROM) or when anaerobes are suspected, differences in clinical practice depending on the obstetrician's preference were observed. However, despite the use of prophylactic antibiotics 98 (64.4%) of our patients developed SSI as compared to 54 who did not receive any antibiotics as prophylaxis. Of 4149 patients enrolled in an observational prospective cohort study that was conducted to directly compare the efficacy of ampicillin *vs.* ceftriaxone as prophylactic antibiotics in preventing post-CS SSIs, 145 (5.4%) patients developed SSI despite receiving either ceftriaxone or ampicillin [39]. This may be explained by the surgical procedure adopted by the surgeon as it has been shown that there is lower risk of wound complications when the subcutaneous thickness of sutures was greater than 2 cm (RR 1.03; 95% CI 0.36–2.76) and with suture skin closure compared with staple skin closure (adjusted OR 0.43; 95% CI 0.23–0.78) [40]. Furthermore, it has been suggested that over, under, improper or misuse of antibiotics occurs in 25–50% of operations [41].

Wound infections represent the most common nosocomial infections in patients undergoing surgery and are often caused by a limited range of opportunist pathogens, namely, *S. aureus*, *E. coli*, *K. pneumoniae* and *Pseudomonas aeruginosa* [33]. However, being a major component of the skin microbiome staphylococci remain the most common organisms responsible for causing SSI [33, 39].

Women opting for a CS for non-medical reasons should be discouraged and informed of the risks of SSI as a complication. Measures taken in the pre-, intra- and post-operative phases can go a long way in reducing the prevalence of SSI post-CS. In the pre-operative phase, measures such as the patient's personal hygiene, antibiotic prophylaxis, proper antiseptic preparation of the surgical site and the use of sterilised instruments all contribute to suppressing post-operative infection.

The limitations of the present study include the lack of evaluation of data related to some of the other pertinent risk factors of SSI such as nutritional status, co-morbidities, skin closure methods and surgical techniques used.

## Conclusion

In view of the increasing rates of CS being performed without a clear medical indication, new practice protocols should be implemented to reduce the rate of cesarean deliveries as CS surgery has a 5–20 times higher risk of post-partum infection as compared to vaginal deliveries [42]. Our study demonstrates that most SSI following CS are detected only after patient's discharge from the hospital. Emergency CS and improper antibiotic prophylaxis are important risk factors in the development of SSI, and given the proliferation of MDR organisms there is an urgent need to implement revised prophylactic antibiotic policy as part of antimicrobial stewardship to reduce infection rates. Further research evaluating all possible risk factors is important for a better understanding of the causes and evolution of SSI post-CS.
